# Targeting *Plasmodium falciparum* Hsp90: Towards Reversing Antimalarial Resistance

**DOI:** 10.3390/pathogens2010033

**Published:** 2013-02-04

**Authors:** Dea Shahinas, Asongna Folefoc, Dylan R. Pillai

**Affiliations:** 1 Department of Laboratory Medicine & Pathobiology, University of Toronto, Canada; E-Mail: dea.shahinas@utoronto.ca (D.S.); 2 Department of Pathology & Laboratory Medicine, The University of Calgary, Calgary, AB, Canada; E-Mail: atfolefo@ucalgary.ca (A.F.); 3 Department of Microbiology, Immunology, and Infectious Diseases, The University of Calgary, Diagnostic & Scientific Centre, Room 1W-416, 9-3535 Research Road NW, Calgary, AB T2L 2K8, Canada

**Keywords:** malaria, hsp90, antimalarial resistance

## Abstract

Malaria continues to exact a great human toll in tropical settings. Antimalarial resistance is rife and the parasite inexorably develops mechanisms to outwit our best drugs, including the now first-line choice, artesunate. Novel strategies to circumvent resistance are needed. Here we detail drug development focusing on heat shock protein 90 and its central role as a chaperone. A growing body of evidence supports the role for Hsp90 inhibitors as adjunctive drugs able to restore susceptibility to traditionally efficacious compounds like chloroquine.

## 1. Introduction

The protozoan parasite *Plasmodium falciparum* is responsible for the most severe form of human malaria and causes a tremendous economic burden [1], leading to at least one million deaths per year, particularly in developing countries where failure to eradicate the anopheline mosquito vector leads to occasional epidemics [2,3]. Approximately 250 million people are infected with malaria worldwide every year, mainly consisting of pregnant women and children under the age of five years [3]. Other species of malaria that infect humans include *P. ovale*, *P. vivax*, *P. malariae*, and *P. knowlesi*. The onset of symptoms takes place at 10–15 days after being bitten, resulting in fever of up to 41.5 °C, chills, headaches, and vomiting [4,5,6,7,8]. If these symptoms are not addressed with chemotherapeutic treatment, the disease may progress to severe malaria [7,8], which consists of severe anemia, acute respiratory failure, hypoglycemia, renal failure, pulmonary edema, seizures, and unarousable coma [8].

Naturally acquired immunity to malaria is slow to develop, is not sterile, and is mostly lost in the absence of continual antigenic exposure [9,10,11]. Individuals raised in malaria-endemic regions eventually become immune to severe malaria and are protected from death [11]. How this immunity is acquired and the duration of the immune memory are not understood. Young children, returning immigrants who have lost previous immunity, and pregnant women are more susceptible to developing severe anemia [8]. Treatment of severe disease with intravenous artemisinin or quinine is an option, but even with this treatment more than 20% of adults, 15% of children, and 50% of pregnant women still die [7]. 

There is no commercially available vaccine to protect against malaria [9,10]. The most promising candidate is the RTS,S vaccine, which consists of the parasite circumsporozoite protein and the hepatitis B surface antigen [12]. It does not provide complete protection, but has demonstrated efficacy against complicated and uncomplicated malaria in children [13,14,15]. This vaccine minimizes the morbidity and mortality of the disease, but does not eliminate the parasite [9]. 

Endeavors to eliminate malaria have raised global interest ever since the 1950s, but have failed because of the resistance of mosquito vectors to insecticides, resistance of the parasites to drugs, socioeconomic problems, and the lack of effective vaccines. Transmission control is one of the main goals of global efforts focusing on increased access to insecticide nets, diagnostic tests, vaccines to prevent disease, and novel therapies [9,16].

Apart from the complexity of the disease, malaria faces a continuous abandonment of drug research and development by the greater part of the pharmaceutical industry due to a lack of profit, which has resulted in a serious shortage in the antimalarial armamentarium [1,6]. A wake-up call for the development of antimalarial strategies was the war in Vietnam, during which large numbers of non-immune soldiers were exposed to chloroquine (CQ)-resistant *P. falciparum* malaria in the 1960s. This allowed the United States Army Research and Development Command and the Walter Reed Army Institute of Research to re-assess valuable old leads for their antimalarial effects [6]. These efforts led to the discovery of two powerful antimalarial drugs: mefloquine and halofantrine [17].

## 2. The Malaria Life Cycle

Before taking its blood meal, the *Anopheles* mosquito releases anticoagulants into the host blood; simultaneously, the injection of malaria sporozoites takes place [18]. The sporozoites travel in the blood until they reach hepatocytes by binding to negatively charged sugars [19]. This is the environment under which the sporozoites grow and replicate their DNA to become multinucleated schizonts that give rise to tens of thousands of merozoites [5]. Merozoites are of minimal size (0.9 × 1.3 µM) and ellipsoidal with a flat-ended apex. They contain an irregularly shaped hemispherical nucleus and a group of secretory vesicles at the apical prominence that are known as rhoptries, micronemes, and dense granules. These vesicles contain the proteins required for invasion [5]. 

**Figure 1:**
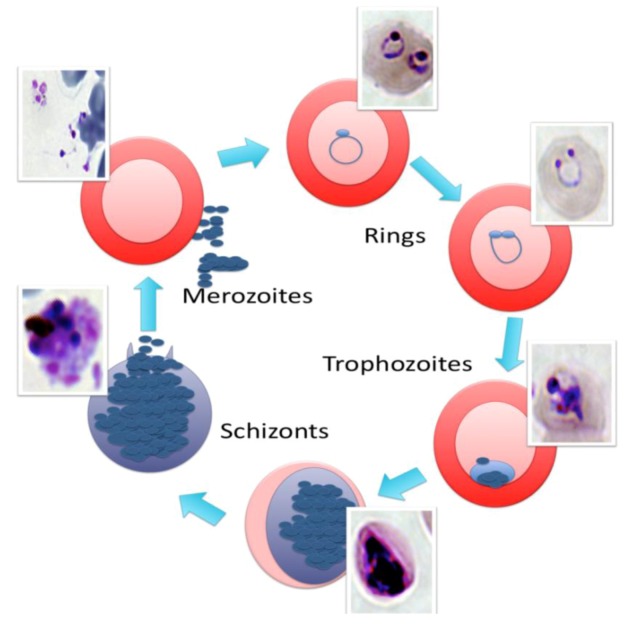
An illustration of the intra-erythrocytic cycle of *Plasmodium falciparum*. The infection of a red blood cell (RBC) during one intra-erythrocytic cycle is depicted. The inset pictures were taken under a light microscope and show the appearance of the parasites at the different stages upon Giemsa staining. One cycle of *P. falciparum* typically takes 48 h to complete*. *

When the merozoites are released from the hepatocytes into the bloodstream, the intra-erythrocytic cycle starts [19]. The erythrocytic cycle is the stage of the parasite life cycle that is responsible for the clinical symptoms of malaria [5], and this cycle can be recapitulated *in vitro*. The stages of the intra-erythrocytic cycle are depicted in Figure 1. For *P. vivax* and *P. ovale* infections, some of the sporozoites develop into dormant parasites called hypnozoites. Their switch into sporozoites and their replication result in relapses that can occur years after the initial infection [7].

The different *Plasmodium* species have different durations to complete one full asexual replication cycle in the blood, e.g., *P. falciparum*, *P. vivax*, and *P. ovale* cycles last 48 h, the *P. malariae* life cycle is 72 h, and the *P. knowlesi *life cycle is 24 h [20,21]. The mouse and rat parasite P. *berghei* has a life cycle that is essentially the same as that of the *P. falciparum* parasite that infects humans, but with differences in the duration of the different stages of the life cycle. The pre-erythrocytic cycle, for example, requires between 42 h and 72 h, while the asexual, intra-erythrocytic cycle lasts between 22 h and 25 h [5]. During *P. falciparum* infection, the addition of the parasite’s erythrocyte membrane binding protein 1 (EMP1) to the surface of the red blood cell (RBC) causes parasitized RBCs to stick to the endothelial cells of blood vessels, avoiding the clearance of these infected cells by the spleen [22] and causing vascular blockage and reduced O_2_ delivery to other organs. The hemolysis leads to anemia and intermittent fever [22]. 

Once *P. falciparum *has successfully invaded a RBC, it spends the first 20–24 h in what is known as the “ring stage” due to the ring-shape appearance of Giemsa-stained parasites under the microscope. During this stage, the parasite feeds on hemoglobin and plasma nutrients, synthesizes ring-stage molecules, and modifies the RBC membrane [5,22]. The ring-shaped parasites are 2–3.7 µm in diameter and discoidal with a biconcave shape. The thicker periphery of the ring contains most of the organelles, including the nucleus [5].

The next stage is the trophozoite stage that appears at 24–36 h after RBC invasion by *P. falciparum *[23]. At this point in its development, the parasite is feeding, growing, and modifying the RBC membrane more than at any other stage [22]. Trophozoites are rounded forms of the parasite that possess a single pigment vacuole with increased basophilia. At this stage, the parasite has lost its biconcave form and rolls into an ellipsoidal or spheroidal shape. The infected RBC starts to develop “knobs,” which consist of dense material from the RBC cytoskeleton [22]. Knobs are first seen in the trophozoite stage, but increase over time and reach a maximum size at the beginning of the schizont period [5]. The trophozoite stage is also characterized by the appearance of two or three cytostomes, which are involved in the formation of many hemoglobin-containing food vacuoles near them. These food vacuoles are internalized and further processing of hemoglobin takes place [5,24]. As the parasite feeds on hemoglobin, it produces toxic heme by-products that it polymerizes into non-toxic hemozoin [24]. As the parasite continues to grow and differentiate, it exports membranes, clefts, small vesicles, and proteins to the surrounding RBC. The molecular details of this export are not well understood [25,26].

Trophozoites replicate their DNA to grow into schizonts [27]. In the schizont stage, the parasite transitions from its trophic activities and cytostomal feeding to maturation. A spindle pole body appears from the nuclear envelope; this is the onset of nuclear division and merozoite formation, and all of the organelles multiply during this phase [27]. The nucleus undergoes four mitotic divisions, resulting in 8–32 nuclei for *P. falciparum*. Synthesized and organized organelles take over the entire area of the RBC. This process of organelle replication continues as the merozoites bud off from the cytoplasm [5,27,28]. 

Once the RBCs infected with schizonts burst, they release as many as 16–32 new merozoites that have the potential to invade fresh RBCs and repeat the intra-erythrocytic cycle [28]. In some cases, the merozoites enter RBCs and do not divide, but differentiate into female and male gametocytes (the crescents that Laveran initially observed). The trigger of this differentiation process is not well understood. When ingested by the mosquito, the male gamete divides into eight flagellated microgametes that escape from the ingested RBC [5]. One microgamete fertilizes the female macrogamete. The resultant zygote is motile and is called an ookinete and has the ability to move through the cells of the stomach wall to form an oocyst. In the oocyst, many threadlike sporozoites are produced through asexual reproduction until the oocyst bursts and the sporozoites are released into the body cavity of the mosquito. They then find their way to the salivary glands so that when the mosquito receives a blood meal, the transmission cycle is complete [5].

## 3. The Emergence of Antimalarial Resistance

Most of the currently available antimalarials have been identified from natural products that exhibit antimalarial activity. The identification of antimalarials such as quinine and artemisinin was serendipitous and involved no rationally identified molecular targets [16,29]. The rest of the antimalarials such as CQ, artesunate, antifolates, and tetracyclines were identified either by their chemical relationship to natural products or from their activity against other infectious pathogens. 

In fact, most of the existing antimalarials have similar modes of action: (1) 4-aminoquinolines (e.g., CQ and amodiaquine) and aryl amino alcohols (e.g., mefloquine, lumefantrine, and quinine) interfere with the formation of the hemozoin crystal and, therefore, the parasite’s ability to deal with toxic heme by-products [16,29]; (2) antifolates (e.g., sulfadoxine, pyrimethamine, and proguanil) disrupt folate metabolism in the parasite [30]; (3) artemisinin and its active derivatives (e.g., artemether, artesunate, artemotil, and dihydroartemisinin) are endoperoxides that interact with reduced heme and modify the parasite’s enzymes and lipids [31]; and (4) antibiotics that interfere with parasite RNA and inhibit protein synthesis (e.g., tetracycline, doxycycline, and clindamycin) [16,29].

Antifolates are among the very few antimalarials with well-defined targets and mechanism of action in the folate biosynthesis pathway, but resistance to these inhibitors, including pyrimethamine and cycloguanil, arose soon after their deployment as antimalarials [16,29]. The mutations in dihydrofolate reductase that generate resistance first appeared in Asia and spread to Africa [16,32]. Apart from the encountered resistance, other issues associated with current antimalarials are their access and severe side effects. Mefloquine is effective in most countries (apart from Southeast Asia), but it causes severe side effects such as seizures, acute psychosis, and anxiety neurosis [7]. Artemisinin is extracted from *Artemisia annua *for the semi-synthetic production of artemisinin derivatives [31]. The content of artemisinin in each plant is between 0.01–0.8% of the dry weight, making it one of the most expensive treatments [16]. 

Resistance has emerged to all existing drugs in Southeast Asia [16]. Multidrug resistance (defined as resistance to more than three drugs) is also common [16]. Causes of antimalarial resistance include incorrect dosing, non-adherence to dosing regimens, poor drug quality, including the dispensing of fraudulent drugs, poor absorption, and misdiagnosis. Resistance develops when parasites undergo point mutations or gene amplification events that provide a fitness advantage, especially under repeated drug exposure [16]. The World Health Organization recommends that cases of uncomplicated *P. falciparum* malaria be treated using artemisinin-based combination therapy (ACT). However, resistance has emerged recently to ACT at the Thai-Cambodia border and may soon be widespread. The two most widely used and cheapest antimalarial drugs, CQ and sulfadoxine-pyrimethamine (SP), have failed at an unprecedented rate in most malaria-endemic regions [16]. Antimalarial resistance has consequently resulted in increased morbidity and mortality from malaria [33]. 

The use of CQ began worldwide in the 1940s. This drug remained the gold standard for the prevention and treatment of uncomplicated malaria for several decades. It was characterized by its rapid parasiticidal action, low cost ($0.2 for a three-day treatment), safety, and widespread availability [5,16]. CQ is active only at the parasite stages that degrade hemoglobin. CQ acts by binding to the heme moieties produced from proteolytically processed hemoglobin and, as such, it interferes with heme detoxification, which takes place inside the digestive vacuole. Once inside the acidic vacuole environment, CQ becomes diprotonated and membrane-impermeant [5,16]. Resistance to CQ was first documented in the 1950s in Colombia and Thailand. By the 1970s, CQ resistance was widespread in Africa, South America, and Asia [5,16]. In 1987, Krogstad *et al*. showed that CQ-resistant (CQR) parasites released pre-accumulated CQ almost 50 times faster than CQ-sensitive (CQS) parasites [34]. In the early 1990s, Wellems *et al*. identified the CQ resistance transporter (PfCRT) by genetic crosses of Dd2 and HB3 clones (CQR and CQS, respectively) [35]. The CQR allele differs by six to eight point mutations from the canonical CQS HB3 *pfcrt *allele [36,37]. 

Analogous to mammalian cells, the gene for the multidrug transporter, *P.* *falciparum *multidrug resistance transporter (*pfmdr1)*, was amplified in some CQR strains. The PfMDR1 protein is also localized on the membrane of the digestive vacuole [5,16]. Point mutations in PfMDR1 are also linked to CQ resistance [5,16]. In spite of the prevalence of CQR *P. falciparum*, CQ is still widely used in sub-Saharan Africa for symptom alleviation due to its low cost [5,16]. 

For quinine, the first reports of decreased *P. falciparum* susceptibility date back to 1908 in Brazil [30]. In 1984, mefloquine was introduced as a monotherapy in Thailand. Five years later, in 1989, the cure rates with mefloquine dropped by >50%, leading to the development of multidrug resistance [16]. Variance in artemisinin susceptibility sometimes correlates with *pfmdr1 *copy number (25-fold 50% inhibitory concentrations (IC_50_s)) [38]. Even though the target of artemisinin derivatives has been postulated to be PfATP6, a calcium transporting ATPase with homology to mammalian SERCA [38,39], polymorphisms in this gene do not always correlate with resistance [16]. These vicious cycles of antimalarial resistance development are perpetuated by the fact that the only affordable treatment options are rapidly losing their therapeutic efficacy.

However, the widespread resistance to CQ and SP, the two lowest cost antimalarials, provides a great incentive for the development of new therapeutic approaches with novel mechanisms of action, particularly in this era in which the understanding of the biochemistry and genome of malaria may aid in the identification of new rationales and mechanistic approaches. White *et al.* [33] argued that the loss of cheap and effective antimalarials to resistance might represent the single most important threat to the health of people in tropical countries. The life of antimalarials such as CQ can be resurrected by combining them with resistance-reversing or sensitizing agents [40,41,42]. 

The ideal antimalarial should be cheap, kill the parasite quickly, be safe, and address the problem of resistance [16,29]. Fast treatment (three-day antimalarial regimen) ensures compliance, avoids resistance, and allows for the rapid clearance of parasites before severe malaria develops. The discovery of antimalarials with new mechanisms of action avoids the development of cross-resistance and provides the option of synergy with existing antimalarials. Many of the newly discovered inhibitors have very short half-lives [16,29], which necessitates their use in combination with a longer-acting drug such as amodiaquine or CQ.

## 5. Heat Shock Protein 90

Heat shock proteins (HSPs) are a class of highly conserved molecular chaperones that facilitate protein folding [43,44]. HSPs function in partnerships in which protein substrates are partially folded by one group of HSPs and then handed over to another group of chaperones before they reach their full functional conformation [43,44]. For example, Hsp70 and Hsp90 play independent chaperone roles, but they exist in a functional partnership ensuring that some peptide substrates are passed from Hsp70 to Hsp90 [45]. An adaptor called Hop (Hsp70-Hsp90 organizing protein) functionally links Hsp70 to Hsp90 [46]. 

Hsp90 is one of the best-studied members of the HSP family, and it is important for normal eukaryotic growth and development. Cytosolic Hsp90 exists in the form of a multichaperone complex and, together with Hsp70 and Hsp60, helps newly synthesized proteins to fold and to modulate the activities of transcription factors and protein kinases [44,46]. It is postulated that Hsp90 serves as a buffer by preventing cellular toxicity caused by misfolded and aggregated proteins in response to stress [47,48,49]. Hsp90 is not involved in primary protein-folding events, but rather in conformationally labile client protein maturation [50,51,52,53]. It provides a compensatory regulatory mechanism that maintains the functional conformation of regulatory proteins, including many that are involved in drug resistance [47,48,54,55]. Inhibition of the broad spectrum of Hsp90 interactions and signaling pathways provides a wide range of anti-disease effects and a decreased likelihood for developing resistance. Several Hsp90 inhibitors are in clinical evaluation for the treatment of various cancers [49,51,56,57,58,59]. 

Pharmacologic inhibition of Hsp90 effectively results in lethality or reversal of the abnormal phenotype [60,61]. Infection, transformation, and neurodegeneration are all characterized by abnormal cell signaling, altered expression levels, and different protein interactions in the cell. In particular, the ATPase activity of Hsp90 is essential for driving the chaperone cycle and directing binding, induction of the active conformation, and release of its client proteins. Inhibition of this ATPase activity at the *N*-terminal ATP-binding domain is an effective approach to inhibit its function and interaction with client proteins [60,61]. Significant similarity exists at the ATP-binding domain between other eukaryotic stress-inducible Hsp90s and *P. falciparum *Hsp90 (PfHsp90) [4,55,62,63,64,65]. 

**Figure 2:**
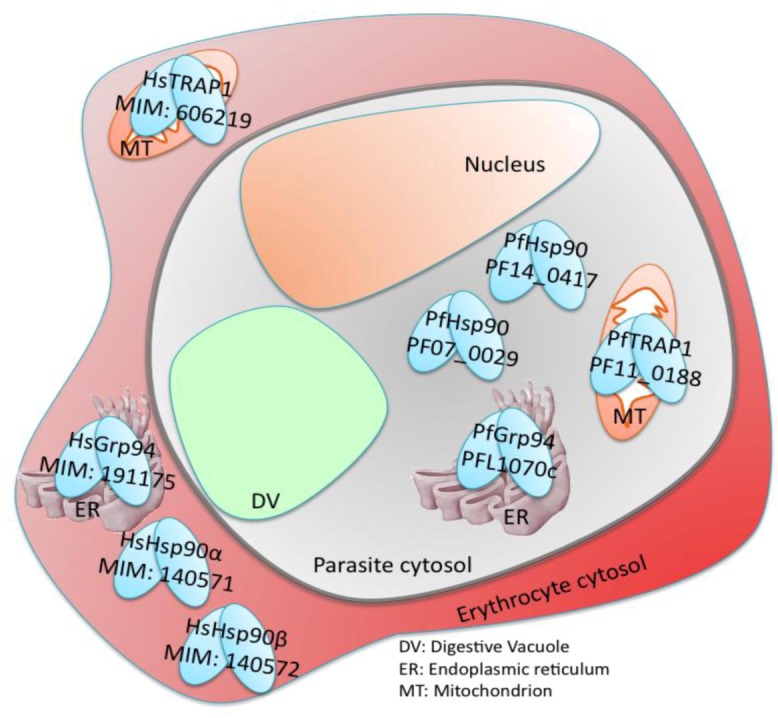
Illustration of the localization of human and malaria Hsp90 paralogs inside a red blood cell (RBC) infected with *P.falciparum*. The focus of this review is on PfHsp90 (PF07_0029), which is the cytosolic isoform with a conserved EEVD motif.

Human and malaria genomes encode four paralogs of Hsp90 (Figure 2). Hsp90α (MIM: 140571) is the human inducible homolog that is over-expressed in cancer cells. Hsp90β (MIM: 140572) is the constitutive form. An additional two paralogs of Hsp90 exist, but unlike Hsp90α and Hsp90β that are cytosolic, the Hsp90 paralog known as TNF receptor-associated protein (TRAP1) (MIM: 606219) is confined to the mitochondria, while Grp94 (MIM: 191175) is localized in the endoplasmic reticulum. These organellar variants are derived separately from the prokaryotic form of Hsp90, known as heat transfer protein G (HTPG). Each of the Hsp90 paralogs folds a distinct set of client proteins [44]. In the human host, Hsp90α and Hsp90β contain an EEVD interaction motif, but for malaria, only one of the cytosolic Hsp90s (PF07_0029) contains the EEVD motif and is known to be induced by stress and highly expressed during the erythrocytic life cycle of the parasite [65]. 

Hsp90 has been exploited as a successful drug target in many cancers, neurodegenerative diseases, and viral and fungal infections due to its activation and mediation of stress-induced interactions in abnormal cells. Hsp90 has a unique Bergerat fold of its ATP binding pocket with an open “lid” domain in the *N*-terminal domain, which can be inhibited competitively and selectively by several small molecules [66]. Since Hsp90 regulates the cell cycle, potentiates drug resistance, and buffers phenotypic variation, many clients of Hsp90 are essential cellular proteins with pathogenic functions that render the inhibition of the Hsp90 pathway lethal to stressed cells, but not to normal cells.

Cowen *et al*. have shown that Hsp90 inhibitors synergize with known antifungals to which the microbe would otherwise be resistant. In fungi [47,67], Hsp90 governs the antimicrobial resistance of agents with different mechanisms of action (e.g., ergosterol and cell wall biosynthesis) [47,67]. Cowen and Lindquist used a titratable [47] Cre-Lox system to examine the role of Hsp90 in potentiating fluconazole resistance. Fluconazole-resistant colonies were present only in strains with high levels of Hsp90. Fluconazole resistance disappeared in strains where Hsp90 expression was reduced by Cre-mediated recombination. The mechanism of resistance could be both acquired and maintained independent of Hsp90 (through mutations in a drug resistance transporter), despite the fact that Hsp90 was shown to be crucial for resistance acquisition. The Hsp90 inhibitors, geldanamycin (GA) and radicicol, reduced fluconazole resistance in *Saccharomyces cerevisiae* strains with mutations in the ergosterol biosynthesis pathway [47]. Thus, this work laid the foundations by showing that Hsp90 mediates the mechanisms that allow cells to cope with abnormal conditions such as drug-induced stress. The most important implication of these findings is that Hsp90 inhibitors have the ability to counter independently evolved drug resistance [47].

Crystal structures have revealed paralog-specific conformational differences in response to ATP or geldanamycin (GA) binding in fungi. For example, while the ATP binding pocket is acidic in Grp94 and yeast Hsp82, the neighboring phosphate-binding regions differ [68,69,70,71]. In yeast Hsp82, this neighboring region is basic and better complements the charge of the nucleotide, while in Grp94, this region is acidic and strongly repels the ligand. Unique differences in the ATPase domains of Hsp90 paralogs account for the specificity of cytosolic Hsp90-targeted inhibitors [68,69,70,71]. Targeting of cytosolic inducible PfHsp90 may result in higher efficacy and therapeutic control. In addition, co-chaperones of Hsp90 vary in distribution across species [72] and consequently, they may also present an opportunity for selective inhibition of the function of Hsp90 in the interacting domains (mainly middle and *C*-terminal domains) [73]. In addition, some co-chaperones of PfHsp90 that have been described such as *P. falciparum* Hsp70-Hsp90 organizing protein (PfHop) show distinct sequence variation in their functional domains compared to the human homologue [74] presenting the possibility of specific inhibition of the PfHsp90-PfHop interaction. However, to date, the selectivity and inhibition of these interacting domains have not been exploited in malaria. 

## 6. Inhibitors of Heat Shock Protein 90

**Figure 3:**
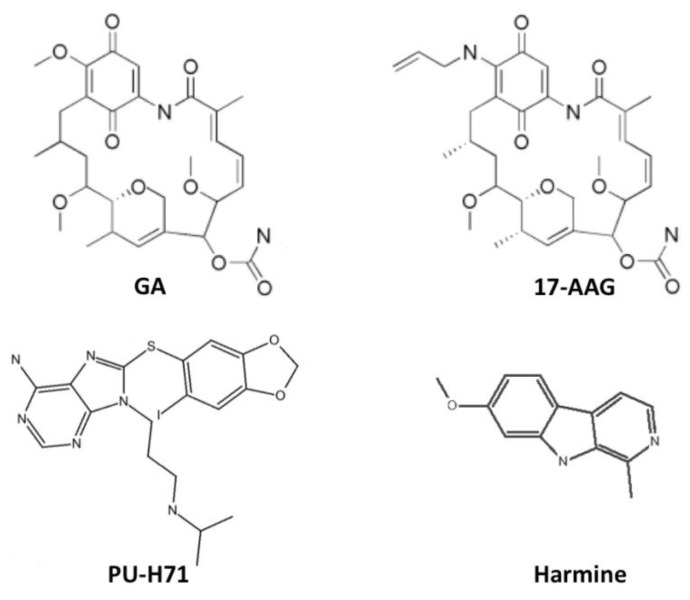
Two-dimensional representation of the chemical structures of the Hsp90 inhibitors discussed in this review: geldanamycin (GA), 17-AAG, PU-H71 and harmine. The structures were generated using Jchempaint [83].

A range of Hsp90 inhibitors that target the *N*-terminal domain have been developed including natural product inhibitors such as geldanamycin (GA) and radicicol, and synthetic inhibitors comprised of purines, pyrazoles, isoxazoles, and other scaffolds [59,60,75]. GA (Figure 3), an ansamycin antibiotic, was the first selective Hsp90 inhibitor shown to bind to Hsp90 and interfere with heterocomplex formation [76]. On inhibition, Hsp90 client proteins cannot attain their active conformation and are degraded by the proteasome [44,77]. Degradation of these proteins leads to growth arrest and apoptosis in several cancer cells. Inhibition of *Brugia pahangi *Hsp90 from endogenous protein extracts has shown that Hsp90 is a selective target in parasites and supports the identification of novel chemotypes with enhanced potency and selectivity [78].

The broad spectrum of Hsp90 interactions and signaling pathways provides a potentially wide range of anti-disease effects and a decreased likelihood for developing resistance. However, Hsp90 inhibition is limited by the compensatory upregulation of Hsp70, which results in counterproductive cytoprotective effects [79]. An additional limitation of the potent Hsp90 inhibitors GA and its analog 17-AAG (Figure 3) is that their clinical use has been precluded by hepatotoxicity, strict dosing limitations, and metabolic and chemical instability [44,71,75]. Radicicol (Rad) is a macrocyclic lactone with anti-Hsp90 activity in cell culture, but it is not stable in serum and, therefore, has no activity *in vivo*. Due to the toxicity caused by GA and its derivatives in animal and human studies, alternative small molecule inhibitors have been sought [44,70,71,75].

Efforts in identifying alternative drugs that inhibit Hsp90 have resulted in the discovery of numerous inhibitors from a variety of scaffolds, which take advantage of the unique shape that ATP assumes when it binds to the Hsp90 ATP binding domain and that show specificity for Hsp90 [75,80,81]. The first members of this ATP-mimetic group were composed of a purine scaffold tethered by a linker to an aryl moiety consisting of the first scaffold drug PU3, which had lower anti-Hsp90 activity than GA and Rad. However, the structure of PU3 was used for extensive chemical modifications in order to enhance its potency, including the introduction of a fluorine at C2 and a sulfide linker [75,80,81,82]. The most potent representative of this group with an attractive pharmacokinetic profile is PU-H71 (MeSH:C526550) [75,80,81,82]. 

## 7. *Plasmodium falciparum* Hsp90

In *P. falciparum*, one cytosolic Hsp90 (PF07_0029, PfHsp90) is known to be induced by stress [44,55] and highly expressed during the erythrocytic life cycle [84]. *PfHsp*90 is essential for the development of the parasite [4,62,65,85]. In a recent study, Pallavi *et al*. also implicated Hsp90 as a drug target against malaria and *Trypanosoma* infections in animal models [64]. Biochemical characterization of full-length PfHsp90 in this study showed that PfHsp90 retained a high level of ATPase activity that was inhibited successfully using GA or 17-AAG. 17-AAG reduced parasite infection load in the *P. berghei* model at the tested dose (50 mg/kg) [64]. 

The crystal structures of the ATP-binding domains of PfHsp90 and human Hsp90 (HsHsp90) reveal a unique Bergerat fold with an open “lid” domain in comparison to other ATPase domains and several unique residues at the drug binding site that can be exploited for drug binding specificity [66,70,71,86,87]. In particular, Val186 of human Hsp90 is replaced by Ile173; PfHsp90 contains Ala38 instead of Ser52 (human Hsp90) and at Lys112, PfHsp90 contains Arg98 [87]. In general, these substitutions result in a more constricted, basic, and hydrophobic pocket in PfHsp90.

PfHsp90 may play a role in the development of drug resistance in the malaria parasite because of its interaction with the CQ resistance-associated protein Cg4 [55]. Hence, PfHsp90 has the potential to not only serve as a drug target but also circumvent drug resistance to conventional antimalarials when used in combination through synergy. Indeed, GA synergizes with calcineurin and cyclosporine A, which target Hsp90-interacting proteins, when tested in tandem for antimalarial activity [62,65]. Furthermore, the parasite interaction network exhibits functional differences from that of *S. cerevisiae* and the human host [55], highlighting the potential of this protein target for the design of specific antimalarial inhibitors.

Heat shock is part of the parasite life cycle when it changes hosts and during the febrile episodes of malaria. Chaperones are very important in responding to this stress and in buffering mechanisms that ensure parasite survival and growth. The increase in temperature activates the PfHsp90 stress response pathway and may facilitate the arousal of new phenotypes by chaperoning mutant client proteins that develop drug resistance, enhance virulence, or cytoadherence properties [62,65]. GA was shown to inhibit growth at an LD_50_ of 200 nM and cause a transition block at the ring stage, suggesting an important role for PfHsp90 in parasite growth. GA inhibition of PfHsp90 disrupted the PfHsp90 complex consisting of PfHsp70, PfPP5, and tubulin, among other proteins [62]. Inhibition of PfHsp90 function in combination with other antimalarials may circumvent the development of drug resistance.

Taken together, the following key factors suggest that PfHsp90 represents a promising target: (1) the antimalarial activity of known cross-species Hsp90 inhibitors such as GA; (2) the essential and multifaceted chaperone function of Hsp90; (3) its potential cross-talk with pathways involved in drug resistance; and (4) the unique structural features in the ATP-binding domain between human and *P. falciparum* Hsp90.

## 8. The PfHsp90 Drug Pipeline

Because parasite drug resistance is increasing due to widely used monotherapy and since heat shock protein 90 plays a major role during invasion of the host by the malaria parasite, the identification and development of antimalarial compounds that target PfHsp90 has recently attracted the attention of several groups. 

Pallavi *et al*. have characterized the role of GA in malaria. Given that PfHsp90 has the highest ATPase activity of all known Hsp90s and has a 30% higher affinity for ATP than HsHsp90, dissociation constants of GA for PfHsp90 and HsHsp90 are found to be 1.05 and 4.4 μM, respectively [64]. This finding is consistent with the results of Wider *et al*. [88] who show that upon exposure of the PfHsp90 and HsHsp90 complemented yeast strains to the two Hsp90 inhibitors radicicol (Rd) and GA, the strain complemented with PfHsp90 is relatively more sensitive to GA than to Rd relative to the strain complemented with HsHsp90. GA and the synthetic analogue, 17-AAG caused 50% inhibition *in vitro* at 25 and 160 nm, respectively, after 48 hours [64]. Even though 17-AAG showed less potency as compared with GA, its IC_50_ value for P. falciparum in culture was comparable with that observed in cancer cell lines. 

Due to the toxicity caused by GA, Rd, and derivatives in animal and human studies, alternative small molecule inhibitors have been sought. To this end, our group performed a robotic high throughput screen (HTS) using 4000 small molecules from a natural compound (Spectrum), pharmacologically active (Lopac), and Food and Drug Administration (FDA) approved drug library (Prestwick) for competitive inhibition of the ATP-binding (GHKL) domain of PfHsp90 [89]. Hits were further screened for selectivity based on differential inhibition of PfHsp90 in comparison to HsHsp90. Three PfHsp90-specific inhibitors were identified: acrisorcin, harmine and 2-Amino-3-phosphono propionic acid (APPA). They showed 50% inhibitory concentrations in the nanomolar range—51.3 nM, 50.3 nM and 60.3 nM, respectively [89]. These hits also demonstrated synergistic activity *in vitro* in the presence of the known antimalarial drug chloroquine. Harmine is a very interesting hit because it belongs to a group of natural metabolites that are the active ingredients of several traditional herbs used to treat malaria in West and Central Africa [90,91,92]. A synergistic effect has been previously observed between extracts that contain harmine and extracts from other medicinal plants used in this region. Several studies have shown potent *in vitro *antimalarial activity of pure compounds or methanolic fractions isolated from these plants [90,93]. We evaluated harmine-PfHsp90 affinity using surface plasmon resonance analysis (dissociation constant Kd of 40 μM. In contrast, the related compound harmalol bound HsHsp90 (Kd of 224 μM) more tightly than PfHsp90 (Kd of 7,010 μM) [94], suggesting that there is specificity of binding across different derivatives of harmine. 

These encouraging results of PfHsp90 inhibition *in vitro *have incited evaluation of antimalarial activity *in vivo *with mouse models of malaria infection. Using Peter's four-day suppressive test, Pallavi *et al.* showed that mice treated with 50 mg/kg 17-AAG significantly reduced parasitemia and had a two-fold prolonged survival time than vehicle-treated control mice [64]. In order to achieve efficacy in cancer models, all 12 Hsp90 inhibitors in clinical trials have been consistently given in a comparable dose range to that administered by Pallavi *et al*., *i.e*., 50–100 mg/kg [95]. For malaria, the therapeutic dose may be higher because treatment is performed over three to four days as opposed to the long-term repeated treatments for cancer models.

Administration of harmine alone (75 mg/kg and 100 mg/kg daily) resulted in a modest effect on parasitemia *in vivo*. However, harmine potentiates the activity of chloroquine *in vivo. *This type of synergy known as potentiation occurs when a compound possesses little or no activity on its own, but it enhances the activity of another active compound [96]. This effect is what our group observed with harmine (75 mg/kg and 100 mg/kg) and chloroquine (5mg/kg) combinations in mice [94]. The synergistic activity of these Hsp90 inhibitors *in vivo *is encouraging for circumventing antimalarial resistance and potentiating the activity of existing antimalarials at subtherapeutic doses. 

## 9. Conclusions

**Figure 4:**
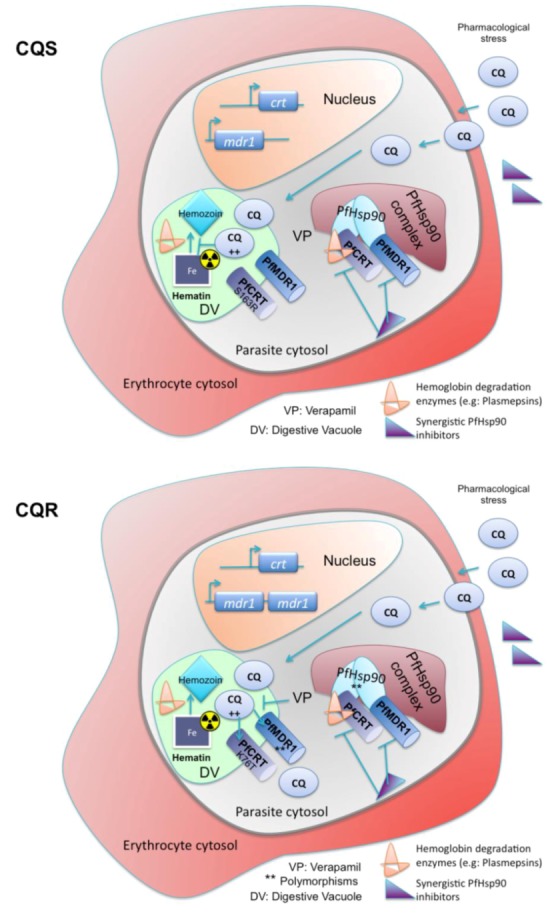
Illustration of the emerging cellular model of PfHsp90 inhibitors and CQ in CQ-sensitive (CQS) and CQ-resistant (CQR) *P.falciparum* infected RBCs. Drug application introduces pharmacological stress, which may result in activation of the PfHsp90 chaperone machinery. CQ accumulation in the digestive vacuole inhibits the detoxification of hemoglobin byproducts in CQS, but not CQR parasites. Inhibition of PfHsp90 chemosensitizes the cells by an arrest of folding clients involved in detoxification, multiple drug resistance and virulence factors. CQS: CQ sensitive CQR: CQ resistant.

In summary, malaria prophylaxis and treatment is challenged by a plastic parasite that is able to generate drug resistance in a short time. The treatment options are limited and novel therapeutic strategies are required. The *in vitro* and *in vivo* data support the role for the Hsp90 inhibitors such as GA, 17-AAG, PU-H71 and harmine, which have a high affinity for the PfHsp90 ATP-binding domain, as being able to act synergistically with CQ. From the study of the PfHsp90 inhibitors synergistic activity with CQ, a complex putative model emerges for both CQS and CQR *P. falciparum* (Figure 4). Under pharmacological stress, the PfHsp90 chaperone machinery is activated. PfHsp90 activity is required for the folding of drug-resistance and virulence associated proteins in the infected cell. Inhibition of PfHsp90 activity hinders trafficking of parasitic proteins to the erythrocyte surface [44], folding of PfCRT and PfMDR1 and hemoglobin byproduct detoxification in both CQS and CQR states.

In CQR parasites, less CQ accumulates in the digestive vacuole due to a critical charge loss mutation of PfCRT (K76T) that permits the transport of charged CQ molecules to the cytosol [97,98,99]. The single amino acid change S163R is thought to block leakage of charged CQ by reintroducing a positive charge to PfCRT, thus restoring CQ sensitivity [99,100,101]. CQR has also been associated with polymorphisms of PfHsp90 that result in the expansion of a pentaglutamate region in the acidic linker domain of the protein [84]. Indeed, another study showed that the region of Chromosome 7 of the *P. falciparum *genome that contains both the *pfcrt *and *pfhsp90 *genes displays reduced recombination activity, but is flanked by recombination hotspots [102], suggesting a recent selective sweep potentially due to extensive CQ pressure in the field. These two independent results attest to selection pressure in conserving these two genes and their association, but simultaneously introducing polymorphisms that are able to confer a fitness advantage due to recombination events in flanking regions. 

However, with this *caveat* in mind, there are significant implications of the Hsp90 inhibitors synergistic activity with conventional antimicrobials when used in combination. Targeting PfHsp90 affords the possibility of developing a synergistic adjunctive therapy with the potential of reversing resistance to existing antimalarials such as CQ. One could envision the development of a chemical genomic catalog of PfHsp90 interactions weighted by the average FIC ratios of the dual targets. The broad spectrum of Hsp90 interactions provides extensive possibilities for adjunctive therapies that decrease the likelihood of developing resistance. A catalog of these interactions will allow for rational selection and circulation of alternative combinations to avoid the prolonged exposure of the parasites to a single therapy. 

Ideally, combination regimens incorporate two novel agents with potent efficacy and similar pharmacokinetic profiles; however, this is not met by any existing combination in use or in development [16,29]. The most successful current combination is ACT, but even patients treated with ACT occasionally suffer from late recrudescences due to the short half-lives of artemisinin derivatives [16,29]. Combination with longer acting drugs is employed with the hope that the potent action of artemisinin will prevent resistance selection to the longer-acting drug. The risk of a single long-acting agent using combination therapy with the PfHsp90 inhibitors GA, 17-AAG and harmine also exists because these drugs have short half-lives. However, the ability of these two drugs to potentiate CQ activity provides strong motivation for their further development. 

Drug development is a laborious and time-consuming process. It is important to start with drugs that have already been investigated for absorption, distribution, and excretion in rodents and humans. Successful candidate drugs need to be easy to manufacture, stable, readily formulated, bioavailable, have an acceptable half-life, and should be safe to administer [103]. For antimalarials, transmission-blocking activity is also a desirable property [16,29]. In the future, the transmission-blocking activity of the PfHsp90 inhibitors and their combinations can be evaluated with *P. falciparum *gametocytes produced *in vitro *[5,6]. Preliminary findings suggest that the PfHsp90 inhibitors such as 17-AAG and harmine are attractive candidates to enable successful drug development due to their promising potency and pharmacological characteristics. These results support the use of PfHsp90 as an antimalarial drug target with the potential of circumventing drug resistance. A drug discovery platform for rational drug combinations in the clinic can be built by integrating proteomic and genomic approaches. 

One limitation of preliminary antimalarial evaluations in general is the use of *in vitro *screens and culturing methods that select for the fittest isolate under laboratory conditions. Multiple drug-resistant and drug-sensitive isolates from around the world have now been culture-adapted and can be obtained from the Malaria Research and Reference Reagent Resource Center. However, all of these isolates have been passaged in multiple laboratories over many years. As such, the biology of these strains has changed and adapted to laboratory life. Therefore, the evaluation of compound activity in these strains can only be predictive. The evaluation of the PfHsp90 inhibitors *in vivo *is encouraging in potentiating the effect of CQ, but their assessment in an endemic malaria site would be paramount for their further clinical evaluation. The attractive research interface with these inhibitors will provide the motivation for their further development. 

## References

[1] Sachs J., Malaney P. (2002). The economic and social burden of malaria. Nature.

[2] Snow R.W., Guerra C.A., Noor A.M., Myint H.Y., Hay S.I. (2005). The global distribution of clinical episodes of plasmodium falciparum malaria. Nature.

[3] Hay S.I., Guerra C.A., Tatem A.J., Noor A.M., Snow R.W. (2004). The global distribution and population at risk of malaria: Past, present, and future. Lancet Infect. Dis..

[4] Acharya P., Kumar R., Tatu U. (2007). Chaperoning a cellular upheaval in malaria: Heat shock proteins in plasmodium falciparum. Mol. Biochem. Parasitol..

[5] Sherman I.W., Sherman I.W. (2005). The life of plasmodium: An overview. Molecular approaches to malaria.

[6] Zak O., Carbon C., Fantin B., Kaminsky R., Sande M.A., O'Reilly T. (1999). Malaria. Handbook of Animal Models of Infection: Experimental Models in Antimicrobial Chemotherapy.

[7] Ashley E., McGready R., Singhasivanon P., Nosten F., Carrara V., Price R. (2006). In vivo sensitivity monitoring of mefloquine monotherapy and artesunate-mefloquine combinations for the treatment of uncomplicated falciparum malaria in thailand in 2003. Trop. Med. Int. Health.

[8] Ndam N.T., Deloron P. (2007). Molecular aspects of plasmodium falciparum infection during pregnancy. J. Biomed. Biotech..

[9] Douradinha B., Doolan D.L. (2011). Harnessing immune responses against plasmodium for rational vaccine design. Trends Para..

[10] Doolan D.L., Dobano C., Baird J.K. (2009). Acquired immunity to malaria. Clin. Micro. Rev..

[11] Langhorne J., Ndungu F.M., Sponaas A.M., Marsh K. (2008). Immunity to malaria: More questions than answers. Nat. Immunol..

[12] Casares S., Brumeanu T.D., Richie T.L. (2010). The rts,s malaria vaccine. Vaccine.

[13] Alonso P.L., Sacarlal J., Aponte J.J., Leach A., Macete E., Milman J., Mandomando I., Spiessens B., Guinovart C., Espasa M. (2004). Efficacy of the rts,s/as02a vaccine against plasmodium falciparum infection and disease in young african children: Randomised controlled trial. Lancet.

[14] Alonso P.L., Sacarlal J., Aponte J.J., Leach A., Macete E., Aide P., Sigauque B., Milman J., Mandomando I., Bassat Q. (2005). Duration of protection with rts,s/as02a malaria vaccine in prevention of plasmodium falciparum disease in mozambican children: Single-blind extended follow-up of a randomised controlled trial. Lancet.

[15] Bejon P., Lusingu J., Olotu A., Leach A., Lievens M., Vekemans J., Mshamu S., Lang T., Gould J., Dubois M.C. (2008). Efficacy of rts,s/as01e vaccine against malaria in children 5 to 17 months of age. New Eng. J. Med..

[16] Fidock D.A., Rosenthal P.J., Croft S.L., Brun R., Nwaka S. (2004). Antimalarial drug discovery: Efficacy models for compound screening. Nat Rev. Drug Disc..

[17] Peters W., Robinson B.L., Ellis D.S. (1987). The chemotherapy of rodent malaria. Xlii. Halofantrine and halofantrine resistance. Ann. Trop. Med. Para..

[18] Tuteja R. (2007). Malaria - an overview. FEBS.

[19] Cowman A.F., Crabb B.S. (2006). Invasion of red blood cells by malaria parasites. Cell.

[20] Bray R.S., Garnham P.C. (1982). The life-cycle of primate malaria parasites. Brit. Med. Bull..

[21] Chin W., Contacos P.G., Coatney G.R., Kimball H.R. (1965). A naturally acquited quotidian-type malaria in man transferable to monkeys. Science.

[22] Bannister L., Mitchell G. (2003). The ins, outs and roundabouts of malaria. Trends Para..

[23] Baumeister S., Winterberg M., Przyborski J.M., Lingelbach K. (2010). The malaria parasite plasmodium falciparum: Cell biological peculiarities and nutritional consequences. Protoplasma.

[24] Francis S.E., Sullivan D.J., Goldberg D.E. (1997). Hemoglobin metabolism in the malaria parasite plasmodium falciparum. Annu. Rev. Micro..

[25] Przyborski J.M., Wickert H., Krohne G., Lanzer M. (2003). Maurer's clefts--a novel secretory organelle?. Mol. Biochem. Para..

[26] Cooke B.M., Mohandas N., Coppel R.L. (2004). Malaria and the red blood cell membrane. Sem. Hematol..

[27] Ben Mamoun C., Gluzman I.Y., Hott C., MacMillan S.K., Amarakone A.S., Anderson D.L., Carlton J.M., Dame J.B., Chakrabarti D., Martin R.K. (2001). Co-ordinated programme of gene expression during asexual intraerythrocytic development of the human malaria parasite plasmodium falciparum revealed by microarray analysis. Mol. Micro..

[28] Koussis K., Withers-Martinez C., Yeoh S., Child M., Hackett F., Knuepfer E., Juliano L., Woehlbier U., Bujard H., Blackman M.J. (2009). A multifunctional serine protease primes the malaria parasite for red blood cell invasion. EMBO.

[29] Fidock D.A. (2010). Drug discovery: Priming the antimalarial pipeline. Nature.

[30] Gregson A., Plowe C.V. (2005). Mechanisms of resistance of malaria parasites to antifolates. Pharm. Rev..

[31] Taylor D.K., Avery T.D., Greatrex B.W., Tiekink E.R., Macreadie I.G., Macreadie P.I., Humphries A.D., Kalkanidis M., Fox E.N., Klonis N. (2004). Novel endoperoxide antimalarials: Synthesis, heme binding, and antimalarial activity. J. Med. Chem..

[32] Imwong M., Pukrittakayamee S., Looareesuwan S., Pasvol G., Poirreiz J., White N.J., Snounou G. (2001). Association of genetic mutations in plasmodium vivax dhfr with resistance to sulfadoxine-pyrimethamine: Geographical and clinical correlates. Antimicrob. Agents Chemother..

[33] White N.J., Nosten F., Looareesuwan S., Watkins W.M., Marsh K., Snow R.W., Kokwaro G., Ouma J., Hien T.T., Molyneux M.E. (1999). Averting a malaria disaster. Lancet.

[34] Krogstad D.J., Gluzman I.Y., Kyle D.E., Oduola A.M., Martin S.K., Milhous W.K., Schlesinger P.H. (1987). Efflux of chloroquine from plasmodium falciparum: Mechanism of chloroquine resistance. Science.

[35] Wellems T.E., Walker-Jonah A., Panton L.J. (1991). Genetic mapping of the chloroquine-resistance locus on plasmodium falciparum chromosome 7. Proc. Nat. Acad. Sci. USA.

[36] Djimde A., Doumbo O.K., Steketee R.W., Plowe C.V. (2001). Application of a molecular marker for surveillance of chloroquine-resistant falciparum malaria. Lancet.

[37] Djimde A., Doumbo O.K., Cortese J.F., Kayentao K., Doumbo S., Diourte Y., Dicko A., Su X.Z., Nomura T., Fidock D.A. (2001). A molecular marker for chloroquine-resistant falciparum malaria. New Eng. J. Med..

[38] Eckstein-Ludwig U., Webb R.J., Van Goethem I.D., East J.M., Lee A.G., Kimura M., O'Neill P.M., Bray P.G., Ward S.A., Krishna S. (2003). Artemisinins target the serca of plasmodium falciparum. Nature.

[39] Uhlemann A.C., Krishna S. (2005). Antimalarial multi-drug resistance in asia: Mechanisms and assessment. Curr. Topics Micro. Immunol..

[40] White N.J. (1998). Preventing antimalarial drug resistance through combinations. Drug Res. Updates.

[41] Crandall I., Charuk J., Kain K.C. (2000). Nonylphenolethoxylates as malarial chloroquine resistance reversal agents. Antimicrob. Agents Chemother..

[42] Pereira M.R., Henrich P.P., Sidhu A.B., Johnson D., Hardink J., Van Deusen J., Lin J., Gore K., O'Brien C., Wele M. (2011). In vivo and in vitro antimalarial properties of azithromycin-chloroquine combinations that include the resistance reversal agent amlodipine. Antimicrob. Agents Chemother..

[43] Pesce E.R., Cockburn I.L., Goble J.L., Stephens L.L., Blatch G.L. (2010). Malaria heat shock proteins: Drug targets that chaperone other drug targets. Infect. Dis. Drug Tartgets.

[44] Shonhai A. (2010). Plasmodial heat shock proteins: Targets for chemotherapy. FEMS Immunol. Med. Micro..

[45] Wegele H., Muller L., Buchner J. (2004). Hsp70 and hsp90--a relay team for protein folding. Rev. Physiol. Biochem. Pharmacol..

[46] Smith D.F., Sullivan W.P., Marion T.N., Zaitsu K., Madden B., McCormick D.J., Toft D.O. (1993). Identification of a 60-kilodalton stress-related protein, p60, which interacts with hsp90 and hsp70. Mol. Cell. Biol..

[47] Cowen L.E., Lindquist S. (2005). Hsp90 potentiates the rapid evolution of new traits: Drug resistance in diverse fungi. Science.

[48] Cowen L.E., Singh S.D., Kohler J.R., Collins C., Zaas A.K., Schell W.A., Aziz H., Mylonakis E., Perfect J.R., Whitesell L. (2009). Harnessing hsp90 function as a powerful, broadly effective therapeutic strategy for fungal infectious disease. Proc. Nat. Acad. Sci. USA.

[49] Marubayashi S., Koppikar P., Taldone T., Abdel-Wahab O., West N., Bhagwat N., Caldas-Lopes E., Ross K.N., Gonen M., Gozman A. (2010). Hsp90 is a therapeutic target in jak2-dependent myeloproliferative neoplasms in mice and humans. J. Clin. Invest..

[50] Mollapour M., Neckers L. (2011). Post-translational modifications of hsp90 and their contributions to chaperone regulation. Biochim. Biophys. Acta.

[51] Moulick K., Ahn J.H., Zong H., Rodina A., Cerchietti L., Gomes DaGama E.M., Caldas-Lopes E., Beebe K., Perna F., Hatzi K. (2011). Affinity-based proteomics reveal cancer-specific networks coordinated by hsp90. Nat. Chem. Bio..

[52] Tatokoro M., Koga F., Yoshida S., Kawakami S., Fujii Y., Neckers L., Kihara K. (2011). Potential role of hsp90 inhibitors in overcoming cisplatin resistance of bladder cancer-initiating cells. Int. J. Cancer.

[53] Wang Y., Trepel J.B., Neckers L.M., Giaccone G. (2010). Sta-9090, a small-molecule hsp90 inhibitor for the potential treatment of cancer. Curr. Opin. Invest. Drugs.

[54] Cowen L.E., Carpenter A.E., Matangkasombut O., Fink G.R., Lindquist S. (2006). Genetic architecture of hsp90-dependent drug resistance. Euk. Cell.

[55] Pavithra S.R., Kumar R., Tatu U. (2007). Systems analysis of chaperone networks in the malarial parasite plasmodium falciparum. PLoS Comp. Bio..

[56] Cerchietti L.C., Hatzi K., Caldas-Lopes E., Yang S.N., Figueroa M.E., Morin R.D., Hirst M., Mendez L., Shaknovich R., Cole P.A. (2010). Bcl6 repression of ep300 in human diffuse large b cell lymphoma cells provides a basis for rational combinatorial therapy. J. Clin. Invest..

[57] Taldone T., Gillan V., Sun W., Rodina A., Patel P., Maitland K., O'Neill K., Chiosis G., Devaney E. (2010). Assay strategies for the discovery and validation of therapeutics targeting brugia pahangi hsp90. PLoS Neg. Trop. Dis..

[58] Taldone T., Gomes-DaGama E.M., Zong H., Sen S., Alpaugh M.L., Zatorska D., Alonso-Sabadell R., Guzman M.L., Chiosis G. (2011). Synthesis of purine-scaffold fluorescent probes for heat shock protein 90 with use in flow cytometry and fluorescence microscopy. Bio. Med. Chem. Let..

[59] Taldone T., Zatorska D., Patel P.D., Zong H., Rodina A., Ahn J.H., Moulick K., Guzman M.L., Chiosis G. (2011). Design, synthesis, and evaluation of small molecule hsp90 probes. Bioorg. Med. Chem..

[60] Jhaveri K., Taldone T., Modi S., Chiosis G. (2011). Advances in the clinical development of heat shock protein 90 (hsp90) inhibitors in cancers. Biochim. Biophys. Acta.

[61] Usmani S.Z., Chiosis G. (2011). Hsp90 inhibitors as therapy for multiple myeloma. Clin. Lymp. Myel. Leuk..

[62] Banumathy G., Singh V., Pavithra S.R., Tatu U. (2003). Heat shock protein 90 function is essential for plasmodium falciparum growth in human erythrocytes. J. Biol. Chem..

[63] Kumar R., Pavithra S.R., Tatu U. (2007). Three-dimensional structure of heat shock protein 90 from plasmodium falciparum: Molecular modelling approach to rational drug design against malaria. J. Biosc..

[64] Pallavi R., Roy N., Nageshan R.K., Talukdar P., Pavithra S.R., Reddy R., Venketesh S., Kumar R., Gupta A.K., Singh R.K. (2010). Heat shock protein 90 as a drug target against protozoan infections: Biochemical characterization of hsp90 from plasmodium falciparum and trypanosoma evansi and evaluation of its inhibitor as a candidate drug. J. Biol. Chem..

[65] Pavithra S.R., Banumathy G., Joy O., Singh V., Tatu U. (2004). Recurrent fever promotes plasmodium falciparum development in human erythrocytes. J. Biol. Chem..

[66] Dutta R., Inouye M. (2000). Ghkl, an emergent atpase/kinase superfamily. Trends Biochem. Sci..

[67] Singh S.D., Robbins N., Zaas A.K., Schell W.A., Perfect J.R., Cowen L.E. (2009). Hsp90 governs echinocandin resistance in the pathogenic yeast candida albicans via calcineurin. PLoS Path..

[68] Dollins D.E., Immormino R.M., Gewirth D.T. (2005). Structure of unliganded grp94, the endoplasmic reticulum hsp90. Basis for nucleotide-induced conformational change. J. Biol. Chem..

[69] Dollins D.E., Warren J.J., Immormino R.M., Gewirth D.T. (2007). Structures of grp94-nucleotide complexes reveal mechanistic differences between the hsp90 chaperones. Mol. Cell.

[70] Immormino R.M., Dollins D.E., Shaffer P.L., Soldano K.L., Walker M.A., Gewirth D.T. (2004). Ligand-induced conformational shift in the n-terminal domain of grp94, an hsp90 chaperone. J. Bio. Chem..

[71] Immormino R.M., Metzger L.E.t., Reardon P.N., Dollins D.E., Blagg B.S., Gewirth D.T. (2009). Different poses for ligand and chaperone in inhibitor-bound hsp90 and grp94: Implications for paralog-specific drug design. J. Mol. Bio..

[72] Johnson J.L., Brown C. (2009). Plasticity of the hsp90 chaperone machine in divergent eukaryotic organisms. Cell Stress Chap..

[73] Shonhai A., Maier A.G., Przyborski J.M., Blatch G.L. (2011). Intracellular protozoan parasites of humans: The role of molecular chaperones in development and pathogenesis. Pro. Pep. Lett..

[74] Gitau G.W., Mandal P., Blatch G.L., Przyborski J., Shonhai A. (2012). Characterisation of the plasmodium falciparum hsp70-hsp90 organising protein (pfhop). Cell Stress Chap..

[75] Chiosis G., Caldas Lopes E., Solit D. (2006). Heat shock protein-90 inhibitors: A chronicle from geldanamycin to today's agents. Curr. Opin. Invest. Drugs.

[76] Whitesell L., Mimnaugh E.G., De Costa B., Myers C.E., Neckers L.M. (1994). Inhibition of heat shock protein hsp90-pp60v-src heteroprotein complex formation by benzoquinone ansamycins: Essential role for stress proteins in oncogenic transformation. Proc. Nat. Acad. Sci. USA.

[77] Chiosis G., Vilenchik M., Kim J., Solit D. (2004). Hsp90: The vulnerable chaperone. Drug Disc. Today.

[78] Taldone T., Gillan V., Sun W., Rodina A., Patel P., Maitland K., O'Neill K., Chiosis G., Devaney E. (2010). Assay strategies for the discovery and validation of therapeutics targeting brugia pahangi hsp90. PLoS Negl. Trop. Dis..

[79] Elo M.A., Kaarniranta K., Helminen H.J., Lammi M.J. (2005). Hsp90 inhibitor geldanamycin increases hsp70 mrna stabilisation but fails to activate hsf1 in cells exposed to hydrostatic pressure. Biochim. Biophys. Acta.

[80] Taldone T., Chiosis G. (2009). Purine-scaffold hsp90 inhibitors. Curr. Topics Med. Chem..

[81] Taldone T., Gozman A., Maharaj R., Chiosis G. (2008). Targeting hsp90: Small-molecule inhibitors and their clinical development. Curr. Opin. Pharmacol..

[82] Caldas-Lopes E., Cerchietti L., Ahn J.H., Clement C.C., Robles A.I., Rodina A., Moulick K., Taldone T., Gozman A., Guo Y. (2009). Hsp90 inhibitor pu-h71, a multimodal inhibitor of malignancy, induces complete responses in triple-negative breast cancer models. Proc. Natl. Acad. Sci. USA.

[83] Krause S., Willighagen E., Steinbeck C. (2000). Jchempaint: Using the collaborative forces of the internet to develop a free editor for 2d chemical structures. Molecules.

[84] Su X.Z., Wellems T.E. (1994). Sequence, transcript characterization and polymorphisms of a plasmodium falciparum gene belonging to the heat-shock protein (hsp) 90 family. Gene.

[85] Banumathy G. (2004). Functional insights into heat shock protein 90 multichaperone complex in plasmodium falciparum. A Thesis submitted for the Degree of Doctor.

[86] Immormino R.M., Kang Y., Chiosis G., Gewirth D.T. (2006). Structural and quantum chemical studies of 8-aryl-sulfanyl adenine class hsp90 inhibitors. J. Med. Chem..

[87] Corbett K.D., Berger J.M. (2010). Structure of the atp-binding domain of plasmodium falciparum hsp90. Proteins.

[88] Wider D., Peli-Gulli M.P., Briand P.A., Tatu U., Picard D. (2009). The complementation of yeast with human or plasmodium falciparum hsp90 confers differential inhibitor sensitivities. Mol. Biochem. Parasitol..

[89] Shahinas D., Liang M., Datti A., Pillai D.R. (2010). A repurposing strategy identifies novel synergistic inhibitors of plasmodium falciparum heat shock protein 90. J. Med. Chem..

[90] Ancolio C., Azas N., Mahiou V., Ollivier E., Di Giorgio C., Keita A., Timon-David P., Balansard G. (2002). Antimalarial activity of extracts and alkaloids isolated from six plants used in traditional medicine in mali and sao tome. Phyto. Res..

[91] Azas N., Laurencin N., Delmas F., Di G.C., Gasquet M., Laget M., Timon-David P. (2002). Synergistic in vitro antimalarial activity of plant extracts used as traditional herbal remedies in Mali. Par. Res..

[92] Fiot J., Sanon S., Azas N., Mahiou V., Jansen O., Angenot L., Balansard G., Ollivier E. (2006). Phytochemical and pharmacological study of roots and leaves of guiera senegalensis j.F. Gmel (combretaceae). J. Ethnopharmacol..

[93] Traore-Keita F., Gasquet M., Di Giorgio C., Ollivier E., Delmas F., Keita A., Doumbo O., Balansard G., Timon-David P. (2000). Antimalarial activity of four plants used in traditional medicine in mali. Phyto. Res..

[94] Shahinas D., Macmullin G., Benedict C., Crandall I., Pillai D.R. (2012). Harmine is a potent antimalarial targeting hsp90 and synergizes with chloroquine and artemisinin. Antimicrob. Agents Chemother..

[95] Biamonte M.A., Van de Water R., Arndt J.W., Scannevin R.H., Perret D., Lee W.C. (2010). Heat shock protein 90: Inhibitors in clinical trials. J. Med. Chem..

[96] Berenbaum M.C. (1989). What is synergy?. Pharmaco. Rev..

[97] Cooper R.A., Hartwig C.L., Ferdig M.T. (2005). Pfcrt is more than the plasmodium falciparum chloroquine resistance gene: A functional and evolutionary perspective. Acta Trop..

[98] Cooper R.A., Ferdig M.T., Su X.Z., Ursos L.M., Mu J., Nomura T., Fujioka H., Fidock D.A., Roepe P.D., Wellems T.E. (2002). Alternative mutations at position 76 of the vacuolar transmembrane protein pfcrt are associated with chloroquine resistance and unique stereospecific quinine and quinidine responses in plasmodium falciparum. Mol. Pharmacol..

[99] Chinappi M., Via A., Marcatili P., Tramontano A. (2010). On the mechanism of chloroquine resistance in plasmodium falciparum. PloS One.

[100] Johnson D.J., Fidock D.A., Mungthin M., Lakshmanan V., Sidhu A.B., Bray P.G., Ward S.A. (2004). Evidence for a central role for pfcrt in conferring plasmodium falciparum resistance to diverse antimalarial agents. Mol. Cell.

[101] Wellems T.E. (2004). Transporter of a malaria catastrophe. Nat. Med..

[102] Mu J., Myers R.A., Jiang H., Liu S., Ricklefs S., Waisberg M., Chotivanich K., Wilairatana P., Krudsood S., White N.J. (2010). Plasmodium falciparum genome-wide scans for positive selection, recombination hot spots and resistance to antimalarial drugs. Nat. Genet..

[103] Leeson P.D., St-Gallay S.A. (2011). The influence of the 'organizational factor' on compound quality in drug discovery. Nat. Rev. Drug Discovery.

